# A Review of Nanotechnology for Treating Dysfunctional Placenta

**DOI:** 10.3389/fbioe.2022.845779

**Published:** 2022-03-24

**Authors:** Huabo Jiang, Li Li, Dan Zhu, Xinyao Zhou, Yongsheng Yu, Qian Zhou, Luming Sun

**Affiliations:** ^1^ Shanghai Key Laboratory of Maternal Fetal Medicine, Department of Fetal Medicine and Prenatal Diagnosis Center, Shanghai First Maternity and Infant Hospital, School of Medicine, Tongji University, Shanghai, China; ^2^ Reproductive Medicine Center, Shuguang Hospital Affiliated to Shanghai University of Traditional Chinese Medicine, Shanghai, China; ^3^ Clinical and Translational Research Center, Shanghai First Maternity and Infant Hospital, School of Medicine, Tongji University, Shanghai, China

**Keywords:** placental dysfunction, nanoparticles, nanotechnology, pregnancy, targeted therapeutic delivery

## Abstract

The placenta plays a significant role during pregnancy. Placental dysfunction contributes to major obstetric complications, such as fetal growth restriction and preeclampsia. Currently, there is no effective treatment for placental dysfunction in the perinatal period, and prophylaxis is often delivered too late, at which point the disease manifestation cannot be prevented. However, with recent integration of nanoscience and medicine to perform elaborate experiments on the human placenta, it is expected that novel and efficient nanotherapies will be developed to resolve the challenge of managing placental dysfunction. The advent of nanomedicine has enabled the safe and targeted delivery of drugs using nanoparticles. These smart nanoparticles can load the necessary therapeutic substances that specifically target the placenta, such as drugs, targeting molecules, and ligands. Packaging multifunctional molecules into specific delivery systems with high targeting ability, diagnosis, and treatment has emerged as a novel theragnostic (both therapeutic and diagnostic) approach. In this review, the authors discuss recent advances in nanotechnology for placental dysfunction treatment. In particular, the authors highlight potential candidate nanoparticle-loaded molecules that target the placenta to improve utero-placental blood flow, and reduce reactive oxygen species and oxidative stress. The authors intend to provide basic insight and understanding of placental dysfunction, potential delivery targets, and recent research on placenta-targeted nanoparticle delivery systems for the potential treatment of placental dysfunction. The authors hope that this review will sensitize the reader for continued exploration of novel nanomedicines.

## Introduction

The placenta is a highly specialized transient organ during pregnancy. Placental dysfunction has been shown to be strongly associated with major obstetric diseases, such as fetal growth restriction (FGR), preeclampsia (PE), preterm premature membrane rupture, preterm labor, late spontaneous abortion, and placental abruption (Brosens et al., 2011). Although significant progress has been made in preventing the disease, current obstetric interventions in clinical obstetric practice are limited for management of the disease, after it has set in (Chappell and David, 2016). The interventions in current practice deal mostly with maternal symptoms or terminating pregnancy when maternal condition deteriorates. According to recent studies, the world population is growing by approximately 80 million people each year, most of whom may experience maternal and neonatal disorders. Over 90% of pregnant women take at least one medication during their perinatal period, either over-the-counter or prescribed. Approximately 10% of pregnant women need medical prescriptions due to serious obstetric complications (Mitchell et al., 2011). The embryo is exceptionally sensitive and systemic toxicity of therapeutic agents may endanger the health of the mother and cause side effects to the fetus. This explains why only a limited number of therapeutic agents are applied for obstetric complications. Therefore, it is important to explore effective therapeutic drugs, seeking a balance between minimizing harmful effects on the fetal compartment and maximizing the dose to mothers.

Nanotechnology is the engineering of structures at the molecular level, often <100 nm in size (Kim et al., 2010). The field is expanding exponentially and is particularly designed to combine therapeutic drugs and deliver them to the targeted organs under controllable conditions, maximizing efficacy while minimizing off-target effects of drugs. In recent years, nanostructures have been widely used to prevent and treat diseases and are expected to become a substantial approach that assists in overcoming the limitations of various diseases. However, advances in targeted nanomedicine in the area of placental dysfunction-related diseases are still in their infancy (Kannan and Kannan, 2017). With rapid development and altered metabolism during gestation, the fetus is more sensitive to external toxic agents and represents one of the most vulnerable subgroups compared with adults. Therefore, fetal safety should be one of the utmost priorities when designing therapeutic approaches for placental dysfunction.

There is increasing interest in these novel treatments, as they improve the phenotype of placental dysfunction, including PE and FGR, where they could be used as therapeutic tools during pregnancy. This review focuses on the application of nanostructures and the advances and safety concerns of nanomedicine therapy for maternal and fetal health in placental dysfunction-related diseases. It also focuses on the use of nanoparticles as vectors that mediate the delivery of medicine to the placenta, and potential interventional targets that may have therapeutic effects in the future.

## Pathophysiology and Clinical Characteristics of PE and Fetal Growth Restriction

PE is a series of maternal hypertensive disorders that affect 5–7% of all pregnancies and remains the major cause of death during the perinatal period worldwide, with >70,000 maternal and 500,000 fetal deaths every year (Rana et al., 2019). The clinical categories and characteristics of PE and FGR are shown in Table 1.

**TABLE 1:** Clinical categories and characteristics of pre-eclampsia and foetal growth restriction.

➢ Pre-eclampsia
➢ classically, new-onset hypertension, ≥20 weeks’ gestation in association with:
➢ i. Proteinuria - ≥ 300 mg per day or protein/creatinine ratio ≥30 mg/mmol (0.3 mg/mg)
➢ ii. Other maternal organ dysfunction including:
➢ Acute kidney injury
➢ Liver involvement
➢ Neurological complications
➢ Haematological complications
➢ iii. Uteroplacental dysfunction
Intrauterine Growth Retardation
➢ Early FGR:
➢ In absence of congenital anomalies
➢ GA < 32 weeks
➢ AC/EFW <3rd centile or UA-AEDF; or AC/EFW <10th centile combined with UtA-PI > 95th centile and/or UA-PI > 95th centile
➢ Late FGR:
➢ In absence of congenital anomalies
➢ GA≥ 32 weeks
➢ AC/EFW <3rd centile Or at least two out of three of the following:
➢ 1. AC/EFW <10th centile
➢ 2. AC/EFW crossing centiles >2 quartiles on growth centiles^a^
➢ 3. CPR <5th centile or UA-PI > 95th centile

a Growth centiles are non-customized centiles. BP, systolic blood pressure; AC, fetal abdominal circumference; AEDF, absent end-diastolic flow; CPR, cerebroplacental ratio; EFW, estimated foetal weight; GA, gestational age; PI, pulsatility index; UA, umbilical artery; UtA, uterine artery.

FGR is defined as a condition in which the fetus does not reach its genetic growth potential. The diagnostic criterion for FGR is an ultrasound-estimated fetal weight (EFW) below the 10th percentile of a specific gestational age (GA). It affects about 3–7% of all pregnancies, has significant short-term and long-term complications, and may adversely affect the quality of life of the fetus (Sharma et al., 2016). According to additional fetal biometric parameters, such as biparietal diameter, head circumference, femur length, and abdominal circumference (AC), FGR can be divided into two categories: symmetrical and asymmetrical. Furthermore, the severity of FGR is determined by EFW (Table 1).

**FIGURE 1:**
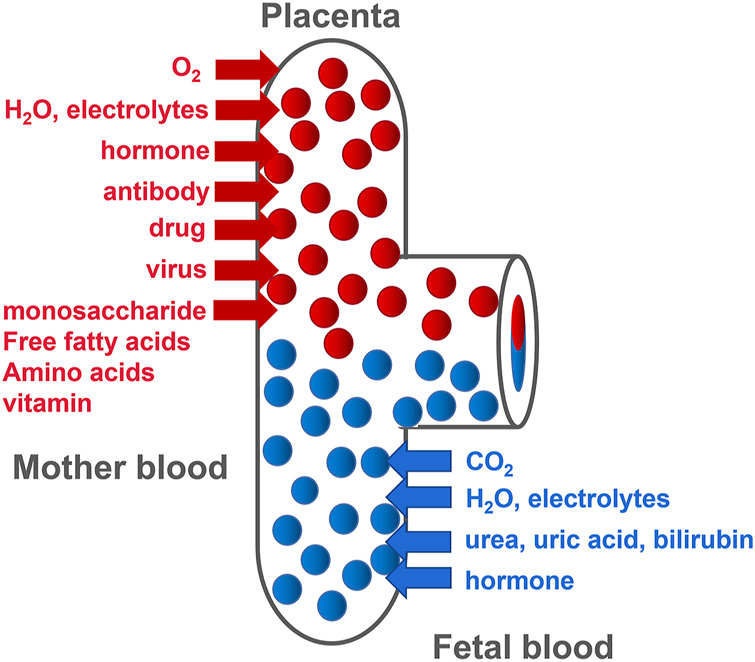
Diagram of material exchange between mother and fetus.

The placenta is mainly composed of mesenchymal cells, trophoblasts, and microvascular endothelial cells. The chorionic membrane is the main functional component of the human placenta. It is composed of fetal capillaries surrounded by multiple trophoblasts (Baker et al., 1993). Trophoblasts are divided into the following three types: progenitor villous cytotrophoblasts (CTBs), extravillous cytotrophoblasts (EVTs), and syncytiotrophoblasts (STBs), which migrate from the villi and invade the maternal decidua. Different trophoblasts play different roles in placental development and function. As progenitor cells of the placenta, CTBs either differentiate to form anchor cell columns, which are responsible for attaching the fetus to the uterine wall, or fuse together to form multinucleated STBs (Genbacev and Miller, 2000). This is due to the further invasiveness of CTBs at the distal end of the cell column where EVTs are formed. These EVTs invade the uterine spiral arteries and convert these blood vessels into a low-resistance state that can transport a large volume of blood to the villus space (Pijnenborg et al., 2006), allowing nutrition, oxygen, maternal-fetal signaling molecules, and drugs to be transported by STB (Figure 1). CTBs fail to deeply invade the uterine wall, causing shallow placentation and incomplete remodeling of the spiral arteries. Inadequate spiral arteriolar remodeling induces insufficient placental perfusion, leading to placental ischemia and maternal syndrome of PE and FGR.

PE and FGR have similar pathophysiological processes and pathogenic factors; both involve uterine placental hypoperfusion, vascular endothelial dysfunction, and abnormal placental invasion (Tal et al., 2010). In normal pregnancy, after forming EVT cells, they invade the spiral arteries such that both the smooth muscle and endothelium of the vessels are eroded (Jauniaux et al., 2018). The pathophysiological process of placental dysfunction in PE, FGR (and other major obstetric syndromes) is mainly abnormal physiological transformation of the endometrial spiral artery, leading to shallow placental implantation and reduced placental blood perfusion (Brosens et al., 2011). PE is a systemic vascular disease, and the basic pathological changes include systemic arteriole spasm, vascular endothelial cell damage, and peroxidative remodeling. Among these, vascular endothelial cell damage can explain various clinical manifestations of the mother, such as hypertension, proteinuria, and edema. One of the main mechanisms underlying the pathophysiology of PE is that placental factors cause maternal vascular endothelial dysfunction, and an increase in placental anti-angiogenic factors is a crucial part of the pathogenesis. Compared with healthy pregnant women, patients with PE have less angiogenesis in the placenta, and the corresponding placental perfusion is reduced, which is consistent with the severity of hypertension (Brosens et al., 2011). At present, there are differing opinions on the pathogenesis of PE. It is generally believed that the PE is caused by a combination of placental, immune, inflammatory, and genetic factors, individually or simultaneously. Despite breakthroughs in our understanding of the etiology of PE, the pathophysiology that triggers the disease remains unclear. Most researchers consider PE to be a two-stage development process, a theory first proposed by Redman (Redman, 1992). The first stage (pre-clinical and symptomless) comprises poor placentation, which is caused by insufficient invasion of cytotrophoblast cells. When cytotrophoblast cells cannot invade the uterine spiral artery normally, the consequence is dysfunctional perfusion of the placenta with oxidative and hemodynamic stress. Excessive antiangiogenic and proinflammatory factors are released into the maternal circulation by the damaged placenta. The second is the clinical expression of the disease stage, when placental factors enter the maternal blood circulatory system, leading to a maternal systemic inflammatory response and systemic vascular endothelial damage, as well as the consequences of placental ischemia. The syndrome is mainly ascribed to diffuse maternal endothelial dysfunction, and the principal clinical manifestations are hypertension and new proteinuria. As the placenta is a unique organ during pregnancy, many pregnancy complications are related to placental structure and dysfunction.

The most common pathological change in FGR is damage to the placental villi. Failure to transform the branches of the placental spiral artery restricts the flow of maternal blood to the villus space, and continuous ischemic and perfusion damages the developing placental villi. Compared with normal pregnancy, the volume and surface area of the fetal FGR placenta decreases significantly, placental thickness increases, the blood vessels in the villi decrease or disappear, the lumen narrows or even becomes occluded, and the percentage of villi, capillaries, and surface area of the villi are all reduced (Erlandsson et al., 2021). Through experiments in guinea pigs, Canas et al. found that FGR is associated with a heterogeneous pro-constrictive vascular remodeling that sets in during pathologic pregnancy to sustain fetal blood redistribution (Cañas et al., 2017). Therefore, FGR not only increases the risk of intrauterine death and related diseases but also affects the growth rate and incidence of cardiovascular disease after birth. In short, the morphology of the FGR placenta is grossly abnormal. This is prominently manifested as the reduction of the surface area and volume of the villi blood vessels, resulting in insufficient placental blood flow, reduced material exchange area, placental hypoxia, malnutrition, and pathological changes around the villi. This results in compression of normal placental tissues, further exacerbating the condition. Abnormal placental morphology eventually leads to placental dysfunction, which adversely affects the fetus in the perinatal period and poses hidden dangers to the health of the fetus after birth.

Studies have found that hypoxia and/or ischemia-reperfusion causes the release of free radicals and inflammatory mediators. This causes cellular stress in STB cells (placental epithelium), resulting in excessive release of numerous factors, leading to exaggerated inflammation, endothelial cell proliferation, and survival defects (Powe et al., 2011). Studies have found that the balance between soluble fms-like tyrosine kinase 1 (sFlt1) and PIGF is of particular clinical importance (Burton et al., 2019; Ives et al., 2020). Excess release of sFlt1 and sENG isolate circulating PIGF and vascular endothelial growth factor (VEGF), thus reducing their bioavailability and subsequently the maternal plasma concentration of these hormones (Cindrova-Davies et al., 2011). This article reviews the pathophysiology of PE and FGR, and proposes several possible therapeutic targets for the treatment of placental dysfunction.

## Overview of Clinical Therapies

Currently, there is no effective clinical therapy for placental conditions resulting in FGR or PE. Physicians often only administer symptomatic treatment or counsel the patient for early induction with premature delivery based on fetal or maternal conditions, which results in a high cost of neonatal intensive care and poor perinatal outcomes (Carr et al., 2017; Morton et al., 2017). Owing to an incomplete understanding of the underlying mechanisms of placental dysfunction in FGR and PE, therapeutic advances have been partially impaired. Current pharmacological treatments have significant similarities between PE and FGR, such as fetal monitoring, perinatal blood pressure control and monitoring, prenatal aspirin therapy, betamethasone for patients aged <34 weeks to mature fetal lung, parenteral magnesium sulfate to protect the brain tissue, preconception counseling, and careful follow-up of postpartum blood pressure. Timely pregnancy termination remains the only definitive treatment. (ACOG, 2013; ACOG, 2019)

## Placental Development

**FIGURE 2:**
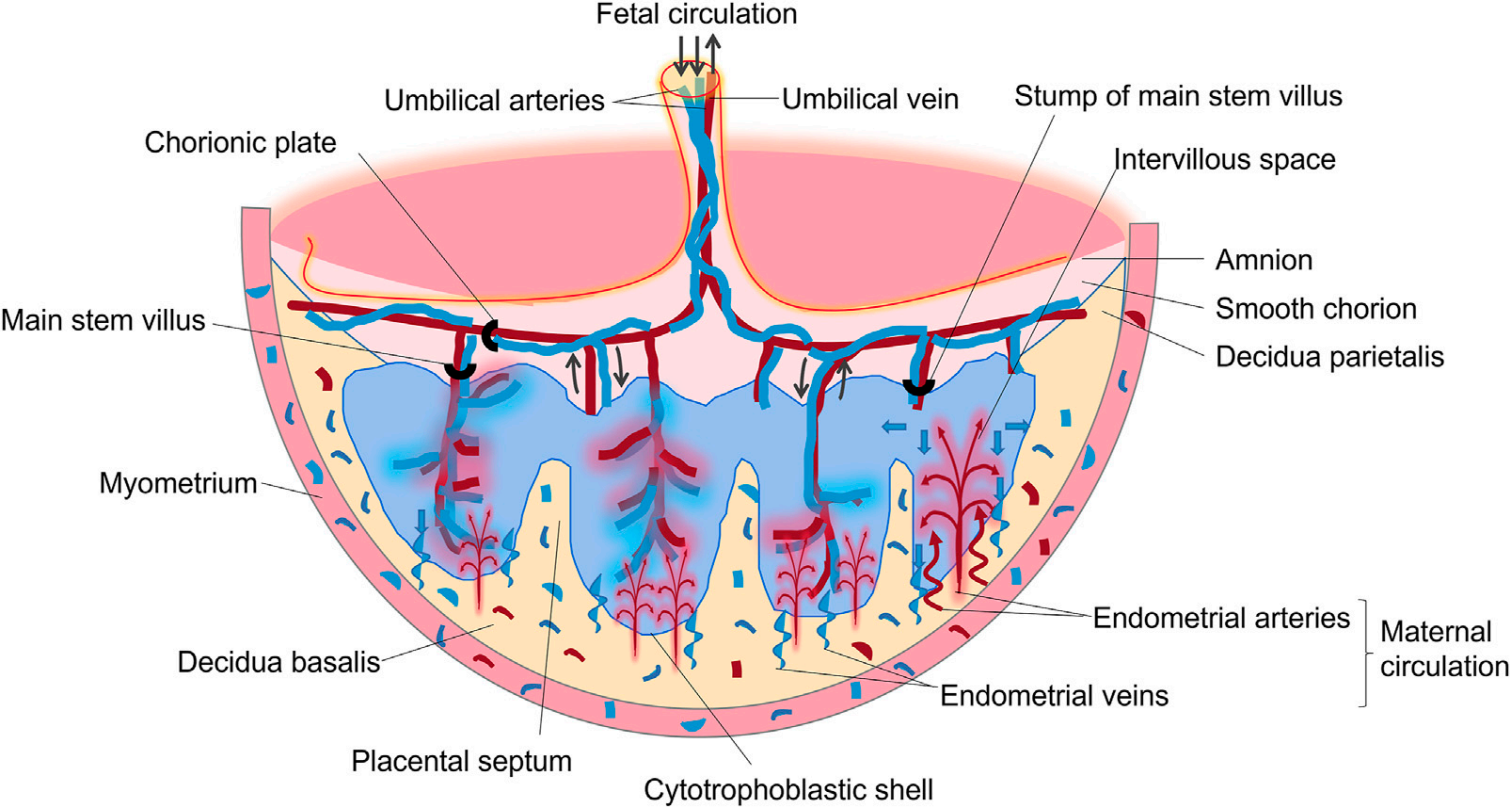
Schematic drawing of a transverse section through a full-term placenta.

The placenta is an important, complex, and special organ between the mother and fetus. It is a critical organ for material exchange, nutrient metabolism, hormone secretion, and protection of the fetus from invasion by microorganisms. The placenta is indispensable for maintaining the normal processes of pregnancy and fetal development. It is composed of the fetal part (villous chorion) and maternal part (decidua), as shown in Figure 2. During pregnancy, the number of villi in contact with the decidua basalis increases rapidly, branching repeatedly, and is called the villous chorion. The placenta is divided by the decidua basalis into independent functional vascular units called cotyledons, which are villous tree-like structures consisting of villous stroma, fetal capillary endothelium, and trophoblast layer. The trophoblast layer is composed of syncytiotrophoblasts, which are derived from the bottom layer of replicating monocytes, called cytotrophoblasts. The trophoblast serves as the placental barrier and is located in the outer layer of the villus tree, which is immersed in maternal blood. As gestation progresses, the number of cytotrophoblasts decreases, resulting in a thinner layer near the end of term.

Approximately 5 days after conception, the embryo is called a blastocyst and contains 50–100 cells. As the blastocyst invades deep into the parenchyma layer of the endometrium, reestablishment of imprinting is underway. Trophoblasts are the outermost cell type of the placenta and are directly exposed to the maternal environment before and after implantation. Precursor monocyte trophoblast cells fuse to form multinucleated syncytial trophoblast cells or acquire a migrating phenotype to become EVTs.

**FIGURE 3:**
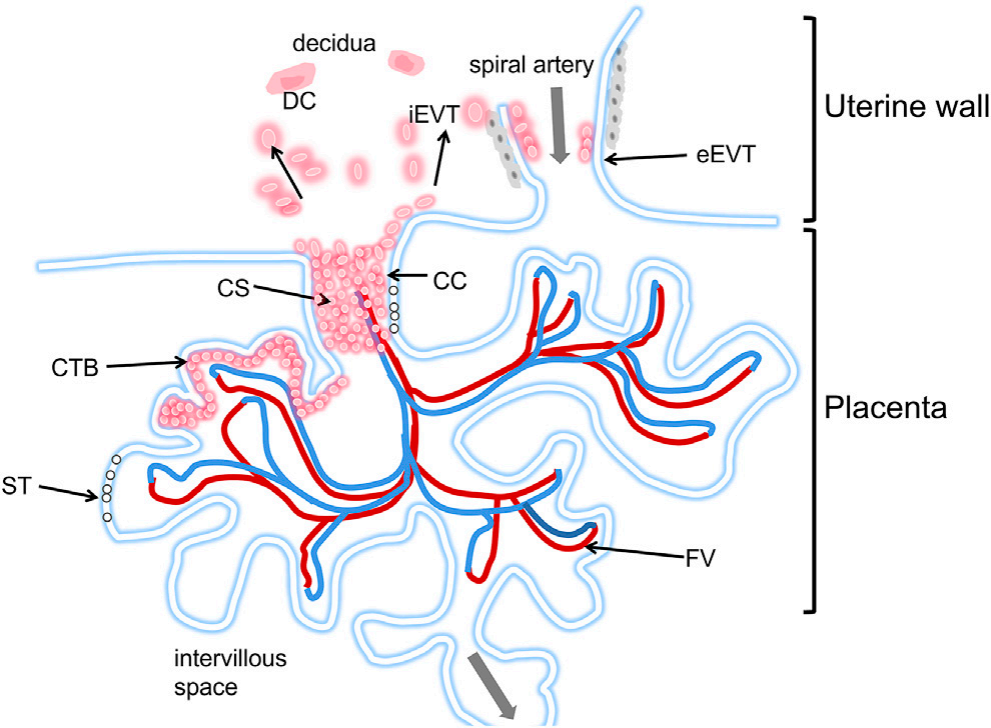
Schematic diagram of the human placental implantation site. The placenta is in the lower part of the picture, Located in the villi space above the uterine wall, and adjacent to a uterine spiral artery. The maternal blood first immersed in the ST layer, and directly below the ST layer is the CTB layer. The blood flows to the fetus through the umbilical cord (see gray arrow). iEVTs are depicted breaking through the CS and invading through the decidua. iEVTs, Invasive interstitial extravillous cytotrophoblasts; ST, Syncytiotrophoblast; CTB, cytotrophoblast; CC, cell column; CS, cytotrophoblast shell; DC, decidual cell; FV, fetal vasculature.

Interactions between the blastocyst and uterine cells trigger the expression of numerous genes in the trophectoderm (Aplin and Ruane, 2017; Ruane et al., 2017) and initiate the development of primary invasive syncytial masses. A cytotrophoblast layer appears on the trophoblast plate. Placental villi appear on day 18 after conception. At this stage, several mononuclear trophoblasts escape from the placenta and begin to invade the maternal interstitium (Figure 3).

In addition to being affected by the development of the placenta itself, circulating maternal substances may also have an impact on placental development. Nutritional ingredients can be transported by trophoblasts to the coelomic cavity and then to the yolk sac. The yolk sac, in turn, transports the metabolism to the embryonic intestine. This pathway is the main nutritional pathway before the formation of placental hemoglobin interface at 11 weeks. However, the specific mechanism of fetal growth defects caused by the lack of histotrophic support for the embryo remains to be explored.

## Potential Delivery Targets

**FIGURE 4:**
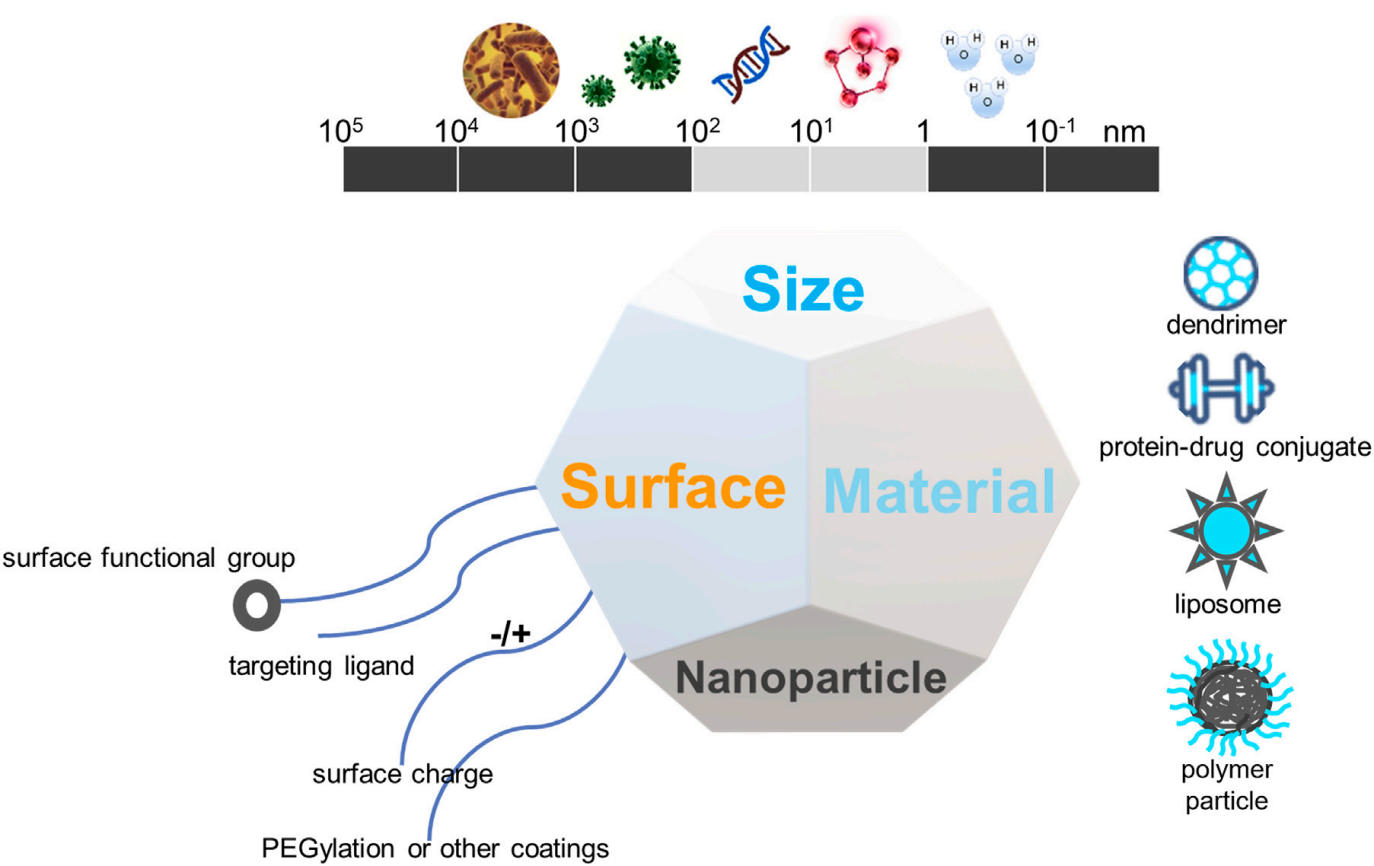
The biophysiochemical properties of nanomaterials for drug delivery in placental dysfunction.

The placenta plays several key roles, including, but not limited to, the delivery of oxygen and nutrients to the fetus and the production of hormones and signals needed to support pregnancy (Lager and Powell, 2012; Napso et al., 2018). The characteristics of placental microvascular endothelial cells are similar to those of trophoblast cells (Troja et al., 2014). This similarity makes cell specificity particularly important, effectively avoiding the off-target effects of nanotherapy or other clinical treatments. Some studies have identified PLAC1 and CyP19a as genes with trophoblast-specific promoters (Rawn and Cross, 2008; Fant et al., 2010). They found that nanoparticles loaded with insulin-like growth factor 1 (IGF-1) molecules could effectively target human trophoblast cell lines. Nanoparticles are nanosized materials (diameter between 1 and 100 nm) that can load multiple drugs or agents. Because of their high surface-area-to-volume ratio and other properties, they can achieve high targeted drug or agent densities. Nanoparticles can also carry the drug within and controlled-release to increase the local drug concentration when tied to the targets. Nanoparticles are divided into different types based on structural differences, including polymeric nanocarriers, polymer conjugates, dendrimers, carbon nanotubes, gold nanocarriers, and lipid-based carriers, such as liposomes and micelles (Figure 4).

These nanoparticles have been used in various applications such as targeted drug delivery, imaging, apoptosis detection, tumor photothermal ablation, and sentinel lymph node localization. Due to the specificity of pregnancy, the application of nanomaterials is strictly limited. In addition to the types of materials, the size of nanomaterials also needs to be carefully designed because the size greatly affects their clearance, cellular uptake, and blood circulation time. For example, nanoparticles >5 nm in size can be quickly cleared by the kidneys. However, nanoparticles between 10 and 100 nm stay longer in circulation and exhibit higher cellular uptake. The type of surface charge also affects nanoparticle absorption (Nel et al., 2009; Blanco et al., 2015). Opsonization can enhance the recognition of NPs by the mononuclear phagocytic system, thereby promoting their uptake in the liver rather than reaching the expected target location. Therefore, to deliver therapeutic agents to targeted placental sites, it is necessary to control the hepatic clearance rate of nanomedicines to develop targeted therapies for the treatment of obstetric complications. Stealth agents, such as polyethylene glycol, can be used to encapsulate nanoparticles to prevent them from binding to blood proteins and have been shown to extend their blood circulation time (Gref et al., 1994; Suk et al., 2016). The safety and distribution of nanoparticles in the placenta are the main problems associated with the clinical use of nanomedicine in pregnancy-related diseases. The lack of knowledge on the transplacental pathways, targeting mechanisms, and their interaction with the placental membranes of NPs further limits the implementation of nanodrugs during pregnancy. In addition, the characteristics of nanoparticles targeting the placenta are not only affected by the physicochemical properties of nanoparticles but also depend on placental maturation and gestational age (Yang et al., 2012). A complete understanding of the placental characteristics facilitates the design of highly targeted nanomaterials and is conducive to the treatment of pregnancy-related diseases. Furthermore, because placental properties vary with pathological conditions, it is necessary to carefully assess the impact of these conditions (Saunders, 2009; Dimasuay et al., 2016). The tunability of nanomedicine has great potential to overcome biological obstacles in targeted drug delivery systems and for the clinical development of nanomedicine to treat maternal and perinatal complications.

Placental pathological conditions affect the interaction between the placenta and nanoparticles and alter gestational age (Mihaly and Morgan, 1983; Wang et al., 2010). Wang (Wang et al., 2010) examined the uptake of drugs at different gestational weeks and found that 63Ni uptake, retention, and transport in the placenta showed a dose-dependent increase. In another study, McIntyre et al. assessed the effects of pathology on the delivery of important amino acids through the placenta and showed reduced delivery of glutamate and glutamine through the placenta in FGR models (McIntyre et al., 2020). As the delivery is limited, the influence of gestational age on nanoparticle targeting efficiency to placenta needs to be further discussed in the future. Since the placenta is immersed in the maternal blood, its main function is to absorb and exchange substances and is thus considered an excellent therapeutic target for dysfunctional placenta treatment. To achieve this specificity, the factors that influence the binding of particles to the placenta need to be recognized and studied. The physicochemical properties of the drug determine its rate of transfer across the placenta, including polarity, molecular weight, size, and lipid solubility (Syme et al., 2004; Evseenko et al., 2006). Most small-molecule drugs (<600 Da) pass through the placenta, mainly via passive diffusion, and are hence subject to placental blood perfusion. Some large proteins such as transthyretin and IgG tend to interact with specific receptors of the syncytial membrane and can be transcytosed across trophoblastic cells into the fetal part (Malek et al., 1995). Contrarily, micromolecules such as nanoparticles typically enter the placenta via other mechanisms, predominantly pinocytosis/endocytosis and phagocytosis (Hillaireau and Couvreur, 2009). Nanoparticles and other macromolecules are absorbed into the placenta, mainly via endocytosis, pinocytosis, and phagocytosis (Conner and Schmid, 2003). However, few studies have been published on the topic of nanoparticles crossing the placenta, and we do not yet fully understand the transplacental absorption and exchange mechanisms of all nanoparticles. Some studies have shown that trophoblasts can absorb polymeric nanoparticles via dynamin-mediated endocytosis. This result supports the ability of NPs to enter the placenta through endocytosis (Bajoria and Contractor, 1997; Menezes et al., 2011).

Systemic small-molecule therapy lacks specificity to the pathological site and can pass through the placenta and reach the fetus, causing adverse effects (Syme et al., 2004). For example, indomethacin is one of the most commonly used tocolytic agents in the clinical treatment of preterm birth because it can easily enter fetal circulation and can also cause side effects, such as intraventricular hemorrhage (Hammers et al., 2015), constriction of the fetal ductus arteriosus (Vermillion et al., 1997; Suarez et al., 2002), and necrotizing enterocolitis (Major et al., 1994; Sood et al., 2011). Targeted delivery aimed at disease-associated cells in the placenta can now open new avenues for developing targeted therapies to address perinatal health. The application of nanotechnology can ensure the delivery of therapeutic drugs to specific cells, thereby preventing harmful side effects in the mother or fetus. Because NPs can be modified to target specific cell populations, nanoloaded drugs can optimize their biodistribution, thereby reducing their side effects. In conclusion, it is vital to select specific targets based on their unique placental structure.

Studies have compared the difference between placental development and cancer, suggesting that the establishment of the placenta may represent controlled cancer (Fidler, 2003; Ferretti et al., 2007; Holtan et al., 2009). Evidence suggests that placental development is similar to genetic and epigenetic regulation of cancer (Lorincz and Schübeler, 2017; Smith et al., 2017). In tumors, p32 has been identified as the main cell surface receptor for the peptide CGKRK (Fogal et al., 2008; Agemy et al., 2013); the same molecule has been shown to be highly expressed in the STB, underlying CTB, vascular endothelium, and villous stroma in the first trimester and term placenta. The expression of p32 was significantly reduced in FGR placentas, suggesting that p32 is important for the proliferation of CTB, as well as the process of FGR (Matos et al., 2014). Calreticulin, a calcium-binding protein in the endoplasmic reticulum (Waisman et al., 1985), plays a significant role in the proliferation, migration, and extracellular matrix degradation of cancer cells (Chiang et al., 2013). The same Calreticulin is overexpressed in PE placental tissues (Shi et al., 2012) and can selectively bind to the synthetic peptide KLGFFKR (Burns et al., 1994). a_v_ integrins are receptors for the homing sequence of iRGD in tumor cells (Sugahara et al., 2009), and act as adhesion molecules to mediate cell signaling and extracellular matrix attachment. The a_v_ integrin is continuously expressed in the mouse placenta throughout pregnancy (Sutherland et al., 1993). Studies have found that integrin a_v_b_3_ is expressed on the surface of the human placenta (Vanderpuye et al., 1991; Zhou et al., 1997). King et al. further developed nanoparticles packaged with FAM-iRGD, which could selectively deliver insulin-like growth factor 2 (IGF-2) to the mouse placenta in a mouse model of FGR. In summary, specific and highly expressed proteins or ligands in the placenta have the potential to be targets for the development of new drugs to treat placental dysfunctions.

## Nanoparticles

Nanomedicine is the application of nanotechnology for the treatment, prevention, monitoring, and control of biological diseases, and has been widely applied in the field of oncology (Peer et al., 2007). The clinical therapeutic effect of nanomaterials also requires precise targets (receptors and/or cells), which can be specifically identified by nanoparticles and are suitable for the delivery system to improve the efficacy of the original drug and minimize side effects. Some of these precise targets include proteins, macrophages, dendritic cells, endothelial cells, and tumor cells (Table 2). When NPs contact and break down their targets, the drug is released to assert its therapeutic function. There are many types of nanomaterials and nanocarriers used for drug transfer with the aim of treating diseases, including liposomes, dendrimers, micelles, polymeric micelles, polymeric nanoparticles, and metallic nanoparticles (Adekiya et al., 2020; Pritchard et al., 2021). In summary, nanomaterials may provide novel treatment options for obstetric medical conditions. A few of these are discussed in the following section, focusing mainly on the three types of nanoparticles applied in the fields of obstetrics and gynecology (Figure 4).

**TABLE 2:** Characteristics of nanoparticles that would be beneficial in treating placental dysfunction.

➢Targeting ability
➢ Target to the mother, placenta or fetus selectively
➢ Reduce risks to the fetus and mother
➢ Increasing efficacy and/or bioavailability of drugs
➢ Lower concentration
➢ Reduce the required dose
➢ Reduce the potential adverse side effects
➢ Modified easily according to the intention
➢ Prevent drug degradation and avoid recognition by the immune systems
➢ Prolong half-life
➢ Target delivery of drugs to the placenta
➢ Encapsulate unstable or insoluble therapeutic agents
➢ Nanoscale properties
➢ Large surface area to volume ratio
➢ Capable to load, carry and deliver drugs
➢ Can be modified and designed to delivery drug through a specific route in the placenta during pregnancy
➢ Reduce dosing of drugs and limit the adverse side effects that the mother or fetus is exposed to

### Liposomes

Liposomal nanoparticles are a subgroup of lipid-based nanoparticles, which are spherical organic engineered vesicles whose central aqueous core is surrounded by a lipid bilayer and can effectively encapsulate macromolecules as well as DNA and siRNA inside the aqueous cores or in lipid membranes, similar to cell membranes. This means that liposomes have better drug distribution and lower systemic toxicity (Park, 2002). These properties make them the most versatile nanocarriers (Bamrungsap et al., 2012; Majzoub and Ewert, 2016; Zylberberg and Matosevic, 2016). Nanoparticles are usually composed of phospholipids and can form unilamellar and multilamellar liposome structures, allowing the transport and delivery of lipophilic, hydrophilic, and hydrophobic drugs and can expand their use by capturing lipophilic and hydrophilic compounds in the same system (Sarfraz et al., 2018). Their stability *in vivo* and *in vitro* can be altered during the synthesis process according to their potential application, which is affected by the nanoparticle size, lipid composition, surface modification, surface charge, and number of lamellae (Sercombe et al., 2015; Fenton et al., 2018; Sedighi et al., 2019). Because liposomes can be rapidly absorbed by the reticuloendothelial system, they are usually surface-modified to enhance delivery and extend their circulation. By conjugating with antibodies or peptides to target nanoparticle therapeutic delivery, it significantly lowers the required therapeutic dose compared to systemic administration. In addition, this conjugation also reduces side effects, and increases tissue targeting specificity. Such conjugation has allowed nanoparticles to be used clinically by loading chemotherapeutic drugs, such as cytarabine and daunorubicin, in treating cancers (Lowis et al., 2006; Khalin et al., 2014; Fonseca-Santos et al., 2015).

### Polymeric Nanoparticles

Polymeric nanoparticles are solid-phase colloidal systems composed of biocompatible and biodegradable polymers, with the additional advantage of the ability to load drugs and proteins without chemical alteration. They can be synthesized from natural, synthetic materials, monomers, or prefabricated polymers (Le and Chen, 2018; Zhang et al., 2019; Brown et al., 2020; He et al., 2020; Zhang et al., 2020), resulting in various structures and functions; these may include dissolving, wrapping, encapsulating, or adsorbing drugs into the polymer matrix, followed by controlled release at the target site. Owing to their simple formulation parameters, they can be formulated to enable precise control of multiple nanoparticle features (Volpatti et al., 2020). These characteristics enable polymeric nanoparticles to deliver various payloads, including hydrophilic and hydrophobic compounds, small molecules, and bio-macromolecules, making polymeric nanoparticles an ideal vehicle for co-delivery applications (Xu et al., 2013; Afsharzadeh et al., 2018; Caldorera-Moore et al., 2019; Jose, 2019; Knight et al., 2019; Strand et al., 2019; Zhang et al., 2020). The most common forms of polymeric nanoparticles are nanocapsules and nanospheres. The former is a cavity surrounded by a polymer film or shell, and the latter is a solid matrix system. Within these two categories, nanoparticles are further divided into polymersomes, dendrimers, and micelles, according to their shapes. Polymersomes have a structure similar to that of liposomes, but are usually composed of synthetic polymer amphiphiles, including poly (lactic acid)-based copolymers, which are difficult to biodegrade and limit their wide clinical application. However, some polymers, including polymer-mediated delivery of chemotherapeutics, are currently used in clinical practice (Rosen et al., 2003; Sabbatini et al., 2004; Duncan, 2006; Etrych et al., 2011) and PEGylated interferon (IFN)-a-2a for hepatitis C (Glue et al., 2000).

### Dendrimers

Dendrimers, are morphologically complex three-dimensional hyperbranched polymers prepared by divergence or polymerization of branched monomers. Dendrimers consist of three regions: a core in the center, inner branches (dendrons), and exterior surface functional groups, each of which is called a generation (G). In the controlled synthesis process, different variables (size, molecular weight, and number of surface groups) gradually increased with increasing G number. For the most familiar poly (amidoamine) (PAMAM) dendrimers, the G0 type has a molecular weight and 1.5 nm diameter, whereas the G7 type has a mass molecular weight of 116,493 and 8.1 nm diameter (Esfand and Tomalia, 2001). Active functional groups enable biomolecules to conjugate to the surface, whereas drugs and DNA/RNA can be encapsulated in the interior. Their size, mass, shape, and surface chemistry can be highly modified and easily customized, making their pharmacokinetics more predictable and controllable (Lee et al., 2005). Similar to liposomes, both are rich in cavities and have a spherical shape with a hydrophobic core and hydrophilic periphery, making them a unique carrier for siRNA delivery (Svenson and Tomalia, 2005; Bawarski et al., 2008). Numerous studies have shown that the active functional groups that exist on the exterior of dendrimers can bind biomolecules or contrast agents to the surface, whereas drugs can be encapsulated in the interior. However, drugs can also be loaded onto the external branching surface of dendrimers, resulting in an extremely high load capacity (Svenson and Tomalia, 2005). Dendrimers can load many kinds of substances but are most commonly used to deliver small molecules and nucleic acids (Xu et al., 2014; Mendes et al., 2017). Several products based on dendrimers are currently being tested in clinical trials. These include theragnostic (therapeutic and diagnostic) agents, molecules used in transfection, contrast agents, topical gels, and charged polymers, such as PAMAM and polyethyleneimine (PEI) (Menjoge et al., 2010a; Kannan et al., 2014; Xu et al., 2014). Although there are currently no dendrimer drugs available for clinical use, they have great potential for clinical translation. Specifically, its utilization can target the delivery of chemotherapeutic drugs, improve the oral route of administration, and enhance intracellular drug delivery (Menjoge et al., 2010a; Myc et al., 2010; Najlah and Demanuele, 2006; Najlah et al., 2007; Wolinsky and Grinstaff, 2008).

## Nanomedicine for the Treatment of Placental Dysfunction

**FIGURE 5:**
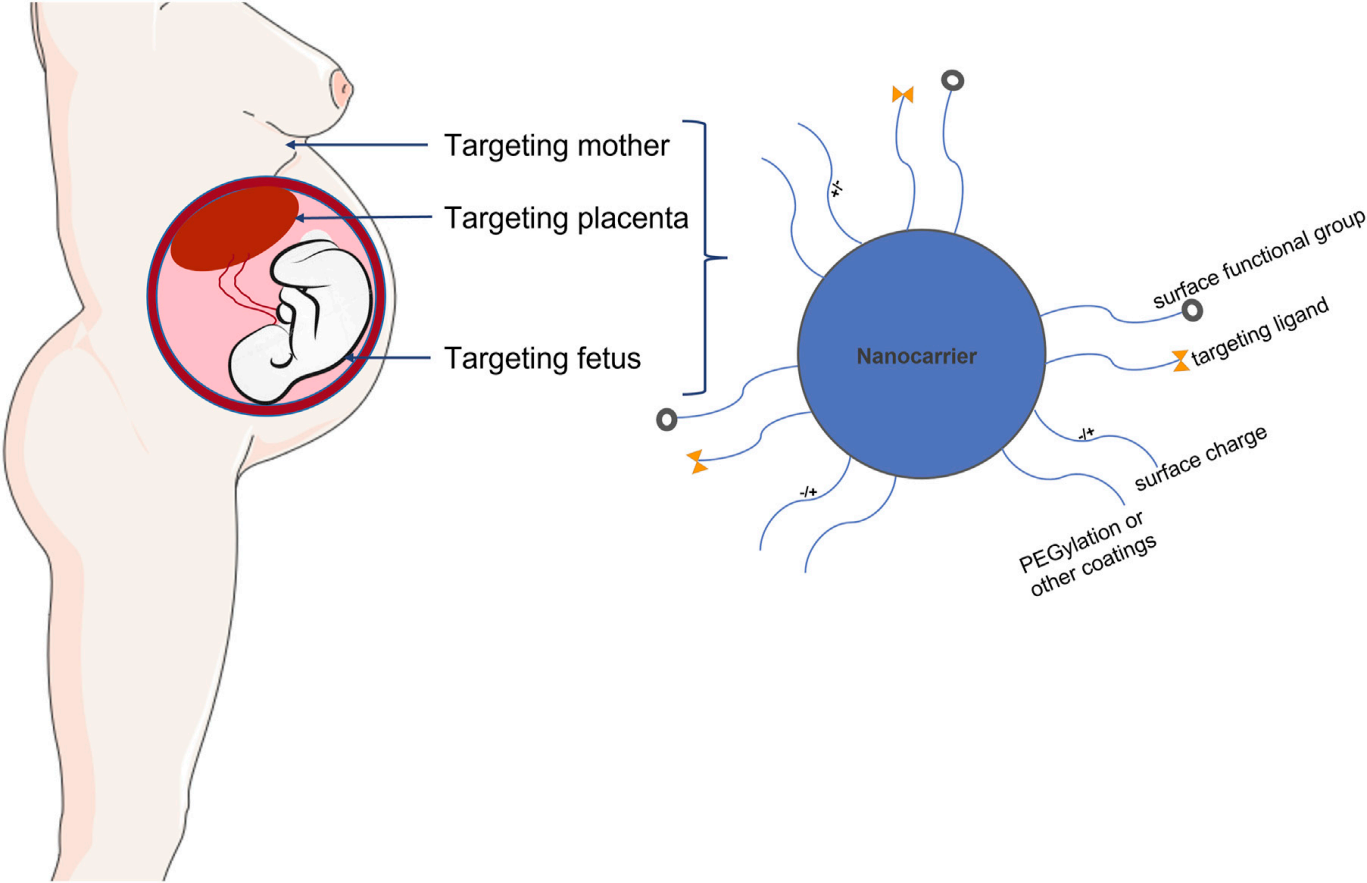
Schematic illustration demonstrating placental targeted drug delivery and nanoparticles applied in pregnancy. Nanoparticle mediated controlled drug and gene delivery specifically targeted to placenta may provide novel avenues for treating placental dysfunction without potential side effects.

In nanomedicine, the development of promising NPs for biomedical applications requires many physical, chemical, biological, and functional properties. The key factor is size. The size and conformation of nanoparticles together determine their trajectory dynamics, which is decisive for nanodrug formulations. Other factors that should be considered include the surface charge of the nanoparticles, encapsulation ability, high drug-loading efficiency, long circulating half-life, minimal systemic toxicity, selective localization, high adhesion in the placental environment, and enhanced internalization and imaging of the placenta through endocytosis. The sustained and controlled release of drugs is an important factor for precise targeted drug delivery of nanodrugs. These characteristics are of great significance for the application of NPs in the diagnosis and treatment of diseases related to placental dysfunction. Most of the aforementioned nanodelivery systems rely on enhanced penetration and retention effects of targeted drug delivery. However, owing to the lack of knowledge regarding highly expressed targets and ligands, the application of nanomedicine in placental dysfunction is limited. In the past few decades, significant progress has been made in understanding the pathophysiology of placental dysfunction and its molecular mechanisms (Table 3). These studies have laid a solid foundation for nanodrug-targeted therapies. As placental dysfunction can lead to many pregnancy complications, the treatment of placental dysfunction can benefit the fetus, thus improving long-term health (Ganguly et al., 2020) (Figure 5). Next, we review the therapeutic options for enhancing placental function, as well as improving the subsequent state of hypoxia and oxidative stress. We also review targets including trophoblasts, placental-specific affinity peptides, and placental growth factor pathways, and targeting placental delivery of miRNA, DNA, mRNA, and siRNA therapies.

**TABLE 3:** Targeted drug delivery systems and human *ex vivo* placenta perfusion model to investigate placenta-NP interactions.

Treatment	Putative mode of action	Test system (s)	Dosing regime	Main outcomes of treatment (compared to appropriate control)	References
CSA targeting PEG-PLA NPs containing siNrf2 and sisFlt-1	inhibit the expression of Nrf2 and sFlt-1 synchronously	PAH model mice	1 mg/kg in T-NPsiNrf2 & sisFTL1 group; 2 mg/kg in T-NPsiNrf2 and T-NPsisFTL1 groups; IV	Decreased circulating Nrf2 and sFlt-1 *in vivo*, and improved pregnancy outcomes	Li et al., (2020a)
PAMAM dendrimer-SiRNA	significantly decreased sFlt1 secretion	HTR-8/SVneo; PE model mice	0.3 mg/kg sFlt1 siRNA at an N/P ratio of 10:1; IV	Decreased circulating sFlt-1 *in vivo*, and improved pregnancy outcomes	Yu et al., (2017)
Tumor homing peptide coated liposomes containing IGF-2	selectively deliver IGF-2 to mouse placenta	placenta-specific P0 knockout mice (P0 mice)	∼ 0.3 mg/kg; IV	Increased mean placental weight and improved fetal weight distribution in healthy and FGR mice respectively	King and Ndifon, (2016)
Peptide coated liposomes containing SE175	significant relaxation of mouse uterine arteries and human placental arteries	endothelial nitric oxide synthase knockout (eNOS−/−) mice	0.44 mg/kg; IV	Increased fetal weight and improved placental efficiency	Cureton et al., (2017)
PEGylated AuNPs	transplacental transfer of nanoparticles in perfused human placenta	human placenta	2.0 × 10^9^–7.9×10^11^ NPs/ml	AuNPs detected in placental tissue; mainly ST and CT layer, not in endothelium of fetal capillaries	Myllynen et al., (2008)
PEGylated AuNPs; Carboxylated AuNPs	different surface modifications (PEGylated versus carboxylated) are taken up and cross the human placental barrier	in a static human *in vitro* co-culture placenta model and the dynamic human *ex vivo* placental perfusion model	25 μg/ml	AuNPs mostly found attached to/in the outer ST layer; PEGylated AuNPs penetrated deeper into the tissue	Aengenheister et al., (2018)
PEGylated AgNPs; Carboxylated AgNPs	AgNPs are taken up and cross the human placenta	in the human *ex vivo* placenta perfusion model	PEGylated AgNPs (2–15 nm; 40 μg/ml) & carboxylated AgNPs	Mass concentration of Ag fraction that accumulated in the placenta	Vidmar et al., (2018)
Fluorescently labeled nonfunctionalized, carboxylated or amine-modified polystyrene beads	the transfer of polystyrene nanoparticles across the human placenta	human placenta	25 μg/ml	Fluorescent amine-modified particles found in the ST and the villous mesenchyme	Grafmueller et al., (2015)
Liposome encapsulated carboxyfluorescein	the low molecular-weight, hydrophilic and polar molecule carboxyfluorescein has been determined across the perfused human term placenta	human term placenta	20 nM	Small (15.2 ± 1.6%)	Bajoria and Contractor, (1997)
				Large (3.0 ± 0.4%)	
				Multilamellar (1.3 ± 0.3%) of initial dose	

Targeting affinity-based peptides was initially applied in the treatment of tumors to deliver therapeutic agents to tumors and related vascular systems (Ruoslahti et al., 2010). As stated in the study, some cell surface antigens are expressed in tumors but absent from healthy tissues. These antigens can bind to circulating ligands, including antibodies and peptides. Therefore, the injection of ligands to which drugs or genes are attached can target the therapeutic agent to be delivered to tumors rather than to normal cells. As previously discussed, placenta and solid tumors have many commonalities, such as rapid cell proliferation, production of growth factors and cytokines, and evasion of immune surveillance. Furthermore, the migration and invasion of placental trophoblast cells in the uterine spiral artery are similar to the invasion and metastasis of cancer cells.

Therefore, to provide a method for drug/gene-specific delivery to the placenta, King and Ndifon, (2016) linked the tumor-homing peptide sequence CGKRK or iRGD to the antigen specifically expressed on the surface of the placenta and bound it to the liposome; specifically, an immunochemistry assay revealed that the peptides could selectively target the segments of endovascular trophoblast lining remodeled arteries and the endothelium of established spiral arteries. The CGKRK-coated peptide also accumulated in the STB layer, but not in the CTB layer, during early gestation and in term human placental explants. In addition, it was found that the tumor-homing peptides CGKRK and iRGD could selectively bind to the human and mouse placenta and did not affect the normal development of the fetus. Liposome nanoparticles coated with these tumor-homing peptides were used as placental targeting ligands and accumulated in the mouse placenta after intravenous injection in pregnant mice. iRGD-coated nanoparticles delivered IGF-2 to the mouse placenta, significantly promoting placental growth in healthy wild-type mice. The average weights of FGR model P0 fetuses is 69% of wild-type birth weight in late gestation (Constancia et al., 2002). Targeted delivery of IGF-2 liposomes effectively increased fetal weight of P0 fetuses (83% wild-type weight), demonstrating the effectiveness of targeted delivery of drugs to the placenta and providing a new method of placental-specific treatment.

Subsequently, the same team further studied another targeting peptide, CNKGLRNK, which specifically binds the vasodilator 2-[[4-[(nitrox)methyl]benzoyl] thio]-benzoic acid methyl ester (SE175) to the placental tissue, as mentioned above. Nitric oxide is packaged to act as a vasodilator and enhance uterine placental perfusion. *In vitro* studies have shown that myography can relax placental blood vessels in both mice and humans. In in vivo studies, intravenous injection of liposomes coated with CNKGLRNK peptide to which the vasodilator SE175 was attached neither promoted placental nor increased fetal weight growth in healthy wild-type mice; however, in a mouse model (nitric oxide synthase knockout mice-eNOS−/−) of FGR, fetuses had a mean weight 13% lower than that of C57BL/6J mice; targeted delivery of SE175 increased mean fetal weight (4%) compared with the control group. In addition to weight gain, the team also found that spiral artery diameter was larger, and the expressions of placental oxidative stress markers, COX-1, COX-2, and 4-hydroxynonenal, were reduced in the placenta (Cureton et al., 2017). Another study (Cureton et al., 2017) showed that the use of specific vascular-targeting peptides to selectively deliver vasodilators to the uterine placental vasculature may provide a promising treatment for FGR caused by impaired uterine placental perfusion.

Other studies have found that microRNAs (miRNAs) affect the growth of the human placenta and fetus (Kang et al., 2015), and that the expression of miRNAs changes in pregnancy complications, including PE and FGR. Therefore, Beards et al. (2017) used a similar peptide-based strategy to study whether targeted placental delivery of miR-675 or miR-145 inhibitor conjugates can relieve these respective miRNA inhibitory effects on the proliferation of the human CTB layer *in vitro* and the growth of the mouse placenta. Therefore, liposomes coated with the peptide CCGKRK were linked to miR-675 or miR-145 inhibitor peptide nucleic acid (PNA) conjugates and injected intravenously into mice or added to human villous placental explants during the first trimester or term pregnancy. Compared with the control group, pregnant mice injected with miR-675 inhibitor or miR-145 inhibitor PNA had increased fetal and placental weights and significantly increased proliferation of CTB, but neither the experimental group nor the control group exerted any effect on litter size or fetal absorption.

These studies have successfully exploited several placental homing peptide-packaged nanoparticles that provide a novel platform as potential therapeutic targets for placental dysfunction. In addition, researchers have also found that other peptides, such as the placental chondroitin sulfate A binding peptide (plCSA) packaged with nanoparticles, can selectively target the trophoblast layer (Zhang et al., 2019). Zhang et al. (2018) showed that plCSA nanoparticles specifically bound to trophoblast cells in mouse and human placental tissue *in vitro*, but not to the fetus, maternal, placental junctional zone, or decidua tissues. Additionally, plCSA NPs were injected intravenously into mice from 6.5 to 14.5 days of pregnancy, and it was found that plCSA NPs containing methotrexate significantly inhibited placental growth and induced apoptosis in the placenta but did not have side effects in maternal tissues. In the control group, animals treated with MTX alone showed severe damage to their maternal tissues, especially the liver and kidneys. In summary, these studies indicated that NPs decorated with a plCSA-binding peptide can be used as a novel placenta-specific therapeutic delivery platform.

Other promising approaches to enhance placental function based on peptide homing include DNA, mRNA, and siRNA therapies to correct the expression of genes that are important in the development of the placenta. IGF1 and IGF2 are critical for achieving appropriate development of the fetal placenta and fetus throughout gestation. Thus, they have been considered as potential targets for intrauterine growth restriction (FGR), which has been a source of interest for many researchers. Jones et al. (2013) demonstrated that overexpression of IGF1 in the placenta improves placental glucose transport in a model of human trophoblasts, thus correcting fetal weight deficits in a mouse model of FGR, and further elaborated the underlying mechanisms of the above effect by enhancing the expression of amino acid transporters (Jones et al., 2014). Abd Ellah et al. (2015) subsequently used a nanostructure delivery system, diblock copolymer (pHPMA-b-pDMAEMA), conjugated with the IGF-1 gene and trophoblast-specific gene promoters of Cyp19a or PLAC1 in a mouse model of FGR and trophoblast cell lines. They found that the offspring weights of experimental group had 20% higher than those in the control group, indicating that the complexes were effective in rescuing fetal growth in a mouse model of the FGR phenotype and significantly increased the expression of placental IGF-1, compared to the same complexes with empty plasmids that do not encode IGF-1. Moreover, Wilson et al. (2020) showed that nanoparticles complexed with IGF-1 and PLAC1 promoters maintained normal fetal growth in an FGR mouse model and in the human placental syncytium. However, it remains to be determined whether it is possible to achieve placental targeting of IGF-1 through the peripheral administration of placental homing peptide-decorated nanoparticles.

As described previously, in most cases of PE and FGR caused by placental dysfunction, the main pathophysiological process is abnormal invasion of the placenta into the uterine wall, which can lead to poor placental perfusion and subsequent hypoxia. Hypoxia in the placenta is assumed to cause STB stress, thus reducing nutrient transport, which, in turn, is a source of antiangiogenic factors for the fetus, such as sFlt1. sFlt1 is an angiogenesis-related factor and current studies have confirmed that it is a clinically specific biomarker for the prevention and diagnosis of PE. Gene therapy has shown great potential in placental dysfunction as demonstrated by Turanov et al. who showed that siRNAs-sFlt1 could effectively reduce circulating and placental levels of sFlt1. This led to alleviation of clinical symptoms, and improvement in pregnancy outcomes in a pregnant mouse model and baboon model of PE, respectively. Gene therapy combined with nanotechnology to regulate sFlt1 expression has been explored as a promising treatment for PE. Yu et al. (2017) found that siRNA-sFlt1-PAMAM dendrimer complexes significantly reduced the secretion of sFlt1, greatly attenuated the symptoms of PE, and improved pregnancy outcomes in a PE mouse model.

Because nanoparticles targeting CSA can specifically deliver drugs to the placenta, Li et al. (2020b) generated carboxyl-polyethylene glycol-poly (D, L-lactide; PEG-PLA) NPs, a new siRNA delivery system, using double-emulsion methods. They complexed placental CSA binding peptide (P-CSA-BP) and PEG-PLA nanoparticles to create a novel delivery system of siRNA-sFlt1 that can specifically target trophoblasts. Their results illustrated a significant decrease in sFlt1 mRNA in the placenta and sFlt1 protein in the serum without any maternal or fetal toxic effects on the utility of T-NPsisFlt1 nanoparticles. The same research group used placenta-targeted PEG-PLA NPs (T-NPNrf2, T-NPNrf2, and sFlt1) to simultaneously downregulate both Nrf2 and sFlt1 in the placenta, and their results showed that inactivation of sFlt1 and Nrf2 alleviated the symptoms of PE and improved pregnancy outcomes. These results suggest that inactivation of sFlt1 and Nrf2 may provide a new therapeutic strategy for PE (Li et al., 2020a).

siRNA by themselves are usually unstable in the bloodstream, thus limited in their ability to reach target tissues and cells. However, when encapsulated by nanoparticles, siRNA complexes have better stability and are easily internalized by the CTB, which minimizes potential side effects to the mother and fetus. Valero et al. (2017) previously confirmed using two *ex vivo* human models, a dual-perfused placenta and suspended villous explants that their liposomes exhibit great potential for the delivery of placental drugs. This was attributed to the fact that they were able to target delivery to the placenta without interfering with fetal circulation. They then applied three different charged states to liposomal formulations to deliver negatively charged siRNA, and the results showed that siRNA complexes were more biocompatible and better internalized by human primary villous CTBs, with a minimized toxicity effect (Valero et al., 2018). Their work highlights that liposome were designed to be used in conjunction with siRNAs and could provide a novel and promising approach to gene therapy in pregnant patients with placental dysfunction-related diseases.

Chronic placental dysfunction also leads to a state of oxidative stress, and accumulating evidence suggests that developing embryos are extremely sensitive to reactive oxygen species (ROS) and oxidative stress during the organogenesis stage. Vafaei-Pour et al. (2018) found protective effects of ceria nanoparticles, showing that the treatment of nanoceria significantly inhibited oxidative stress and pathological changes in embryos. Therefore, systematic administration of antioxidant therapy can attenuate pregnancy complications. Phillips and Scott, (2017) investigated the maternal injection of a non-targeted c-PGA-Phe polymeric nanoparticle-bound antioxidant mitochondrial antioxidant (MitoQ) *in vitro* and *in vivo*. In a hypoxic pregnant rat model, exposure to hypoxia resulted in decreased birth weight. MitoQ combined with nanoparticles (concentrated in the CTB) rescued 60% of the deficit and reduced oxidative stress in the placenta but did not affect the weight of the placenta or fetus. More importantly, nanoparticle complexes were not detected in fetal thoracic, abdominal, or brain tissues. These results demonstrate that antioxidant therapy is a promising candidate for the treatment of placental dysfunction. Targeted delivery of hemoglobin (Hb) via liposomes (hemoglobin vesicles) was also developed as an artificial oxygen carrier for the treatment of fetal hypoxia, and using a model of pregnant rats, they found that there were no adverse effects on fetal development and the pregnant mother, but studies have found that there is the deposition of liposomes in the fetus. This safety study of Hb vesicles during pregnancy may contribute to a novel clinical treatment for placental dysfunction caused by fetal hypoxia. However, the possible clinical effects of liposome deposition require further investigation.

Infection is also one of the causative factors of FGR. Genital infections, both bacterial and viral infections, required urgent treatment during pregnancy. A study evaluated the transplacental kinetics biodistribution and transfer of PAMAM dendrimers and conjugates *ex vivo* across the human placenta model and found that the maternal side placental perfusate was 18-fold higher than the fetal side, indicating that the PAMAM dendrimers exhibited a low transplacental rate (Menjoge et al., 2011). Another study used an *ex vivo* model to evaluate the same PAMAM dendrimers for intravaginal application to treat the ascending genital infections in pregnant women. The results showed that the dendrimers exhibited a low transplacental rate (<3%) compared with almost 50% in the control group (Menjoge et al., 2010b). These results demonstrate that the use of dendrimers in combination with drugs can effectively prevent them from crossing human fetal membranes when administered intravaginally, and thus may provide a new way to selectively deliver therapeutic drugs to the mother, thereby reducing fetal exposure risks. Collectively, the studies outlined above clearly demonstrate the opportunity to exploit novel targeting NPs to deliver therapeutics to the placenta and provide platforms for the development of placenta-specific therapeutics, including gene delivery.

## Discussion

As discussed in this review, nanotechnology presents an exciting opportunity to improve many aspects of our lives and is believed to hold immense potential for treating placental dysfunction, reducing the risks to the mother and fetus. There is an urgent need for novel treatments of placental dysfunction-related diseases. Nanoparticles targeted to the placenta may offer noninvasive options for treating placental dysfunction-related diseases, such as PE and FGR. Specifically, the targeted delivery of therapeutic molecules to the dysfunctional placenta may provide opportunities to treat serious obstetric complications. This review highlights the application of nanotechnology, and the advances and safety concerns of nanomedicine therapy for maternal and fetal health in the phenotype of placental dysfunction. However, it is essential that any associated risks be fully assessed before the field develops too far. Fundamental to developing a comprehensive understanding of the risks of nanoparticles in this area is the evaluation of interaction of NPs with biological barriers, which dictate access to the whole organism and specific organs. Recent advances in targeted delivery strategies have stimulated our interest and broadened our horizons. Hence, our research team aimed to prepare nanodrugs specifically targeting the placenta to provide potential treatments for placental dysfunction.

In summary, the following scientific issues must be resolved in the field of nanoparticle research: 1) Optimal nanoparticle design. To achieve their diagnostic and therapeutic functions, nanoparticles must first be able to break through the physiological and cellular barriers; therefore, they must reach the target tissue and cells at a certain concentration, thus improving the therapeutic effect and reducing side effects on the mother and fetus. Achieving this targeted delivery process through the optimization and innovative structural design of nanoparticles and further increasing the cumulative dose in the placenta are important challenges for future research. 2) Nanoparticles have a complex relationship between structure and function. In addition, they may come in different material characteristics (size, shape, charge, and composition), and may exhibit different physiological environments, that may present different toxicological results in diseases. Thus, it is difficult to approve nanoformulations. To promote the application of NPs, the safety and effectiveness of clinical requirements must be coordinated with the complexity of NPs to promote the development of standard methods for the characterization and preparation of nanomaterials.
